# Practical Departments

**Published:** 1897-02-06

**Authors:** 


### PRACTICAL DEPARTMENTS.

### THE "VICTOR GEM" INVALID TABLE.

This invalid table, designed by Millard Bros., 123, Houndsditch, E.C., has many good points to recommend it for use in the sick room. The table proper is secured to an iron support, sliding into an upright slotted iron frame, controlled by a small hand wheel, which by a turn fixes it in position. The table can be swung round so as to bring the main support more under the centre, when it is required for other than bedside purposes. Of course the height can be regulated, and that with ease, by another small hand wheel. The foot support is particularly steady, consisting of four braces or rests, two short ones extending laterally and two long ones at an oblique angle, with the object of strengthening it at the point of greatest resistance. There are castors, so the table moves smoothly and easily, and it is far firmer on its feet than most tables of this description. The price is 30s.

